# Supplementary material for: Specific Alcohol-Responsive Photonic Crystal Sensors Based on Host-Guest Recognition
Source: Gels. 2023 Jan 18;9(2):83. doi: 10.3390/gels9020083 (PMC9957353; doi:10.3390/gels9020083)
Supplement: Supplementary file 1 [file gels-09-00083-s001.zip › gels-2152412-supplementary.pdf]

The supporting information for

Specific alcohols-responsive photonic crystals sensors based on Host-Guest Recognition

Xiaolu Cai [1], Xiaojing Zhang [2], Jing Fan [1], Wenxiang Zheng [1], Tianyi Zhang [1], Lili Qiu [1,*] and Zihui Meng [1,*]

1 School of Chemistry and Chemical Engineering, Beijing Institute of Technology, Beijing 100081, China
2 Quality Control Center Department, Sinosteel Luoyang Institute of Refractories Research Co. Ltd, Luoyang 471039, China
* Correspondence: qiulili@bit.edu.cn(L.Q.); mengzh@bit.edu.cn(Z.M.)

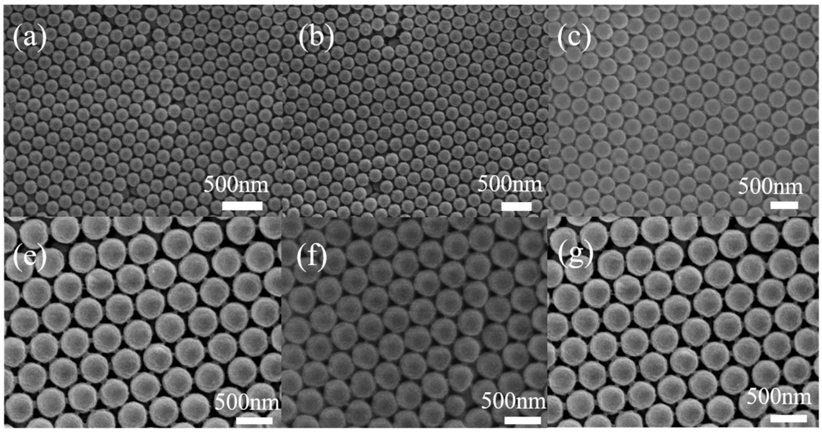

Figure S1. SEM images of PMMA arrays with diameters of (a)165 nm; (b)180 nm; (c)210 nm; (d)225 nm; (e)240 nm and (f)255 nm.

Table S1. Hydrogel formulation optimization table.

number	A-β-CD（g）	AM (g)	BIS (g)	ultrapurewater (mL)	DEAP (μL)
1	0.40	1.60	0.02	8	5
2	0.80	1.60	0.02	8	5
3	1.20	1.60	0.02	8	5
4	1.60	1.60	0.02	8	5
5	2.00	1.60	0.02	8	5
6	1.20	1.60	0.02	8	5
7	1.20	1.60	0.04	8	5
8	1.20	1.60	0.06	8	5
9	1.20	1.60	0.08	8	5
10	1.20	1.60	0.10	8	5

BIS was used as an auxiliary cross-linking agent to tune the binding of hydrogels to PMMA arrays. When the BIS content was too low, the hydrogels and the arrays were not tightly combined. When the BIS content was too high, the degree of cross-linking of the hydrogel was too high, and the reflection peak of the PCs was beyond the visible light range. Therefore, the content of BIS was set at 0.06 g.
